# Application of the C-S-H Phase Nucleating Agents to Improve the Performance of Sustainable Concrete Composites Containing Fly Ash for Use in the Precast Concrete Industry

**DOI:** 10.3390/ma14216514

**Published:** 2021-10-29

**Authors:** Grzegorz Ludwik Golewski, Bartosz Szostak

**Affiliations:** 1 Department of Structural Engineering, Faculty of Civil Engineering and Architecture, Lublin University of Technology, Nadbystrzycka 40 Str, 20-618 Lublin, Poland; 2 Department of Conservation of Built Heritage, Faculty of Civil Engineering and Architecture, Lublin University of Technology, Nadbystrzycka 40 Str, 20-618 Lublin, Poland; b.szostak@pollub.pl

**Keywords:** sustainable precast concrete, prefabrication, siliceous fly ash, nano-admixture, mechanical properties, microstructure

## Abstract

Siliceous fly ash (FA) is the main additive to currently produced concretes. The utilization of this industrial waste carries an evident pro-ecological factor. In addition, such actions have a positive effect on the structure and mechanical parameters of mature concrete. Unfortunately, the problem of using FA as a Portland cement replacement is that it significantly reduces the performance of concretes in the early stages of their curing. This limits the possibility of using this type of concrete, e.g., in prefabrication, where it is required to obtain high-strength composites after short periods of curing. In order to minimize these negative effects, this research was undertaken to increase the early strength of concretes with FA through the application of a specifically formulated chemical nano-admixture (NA) in the form of seeds of the C-S-H phase. The NA was used to accelerate the strength growth in concretes. Therefore, this paper presents results of tests of modified concretes both with the addition of FA and with innovative NA. The analyses were carried out based on the results of the macroscopic and microstructural tests in five time periods, i.e., after 4, 8, 12, 24 and 72 h. The results of tests carried out with the use of NA clearly indicate the possibility of using FA in a wide range of management areas in sustainable concrete prefabrication.

## 1. Introduction

In the modern technology of concrete composites, there is great interest among scientists and practical engineers concerning the possibility of modifying the microstructure of cement-based materials by using chemically active mineral additives. These include natural pozzolans, siliceous and calceorous fly ashes, silica fume, granulated blast furnace slag, lime powder, nano-additives and other materials to replace the cement binder in the composition of the concrete mix [1,2,3,4,5,6,7,8,9,10,11]. These additional components that are part of modern cement matrix composites are referred to as Supplementary Cementitious Materials (SCMs) [12,13,14,15].

However, due to the fact that, in the vast majority of countries in the world, energy generated for industrial and domestic needs is still produced by burning hard coal, siliceous fly ash (FA), which is a by-product of these processes, is the main additive to currently produced concrete composites [16,17,18,19,20]. This is evidenced, among other aspects, by the amount of these by-products generated annually, at almost one billion tons [21].

Therefore, there is an important problem regarding FA management in such a way that they do not adversely affect the environment because, in some respects, they are hazardous materials [22].

In addition, such measures result in a reduction in the basic binder used in the production of concrete composites, i.e., ordinary Portland cement (OPC), in the composition of the concrete mix. Consequently, this results in:

- lower costs of producing such materials [23];
- reduced consumption of thermal and electric energy [24];
- a marked reduction in the emission of harmful greenhouse gases [24].

Considering the above aspects, it must be stated that the utilization of FA also carries an evident pro-ecological factor [25,26,27,28,29,30,31].

Among other aspects, the sustainable precast concrete industry benefits from the use of FA in concrete technology. According to [32], the manufacture of prefabricated building materials containing binding products such as ettringite and calcium silicate hydrate, C-S-H (CSH), can provide, in addition to other well-defined industrial activities, the opportunity to use wastes and by-products as raw materials, thus contributing to the further preservation of natural resources and the protection of the environment. Therefore, intensive research is being carried out around the world to effectively use industrial waste such as FA in prefabricated structures [33,34,35].

Furthermore, the benefits associated with the implementation of FA in the composition of the concrete mix are also related to their positive influence on the processes correlated with the formation of a compact structure in the concrete composites. This is associated with the fact that, during the formation of cement matrix bonds in concrete, the Portland clinker minerals such as allite (C_3_S) and belite (C_2_S)—which constitute approximately 75% of the weight of the basic binder, i.e., OPC—form calcium hydrosilicates and a large amount of calcium hydroxide (CH). Free lime, due to its low strength, low stability and high brittleness, leads to the reduced strength and fracture toughness of concrete and deterioration of its durability [36]. The addition of a sufficient amount of fine-grained pozzolanic additive in the form of FAs that contain active silica (SiO_2_) causes a gradual reaction with CH, consequently forming significant amounts of the CSH phase. As a result, there is not only an improvement in the quality of the hydrates, but also quantitative changes in the structure of the cement matrix. Among other aspects, the following are observed: an overall reduction in the porosity of the material, a change in the pore structure of the paste and a change in the amount of heat released during its hydration [37,38,39]. These processes consequently improve the properties of the hardened concrete composite during the standard period and beyond [40,41]. In the case of composites made on the basis of a cementitious matrix containing FA, among other aspects, a marked reduction in inter-material cracking and increased fracture toughness are observed [42]. Consequently, this leads to the improved durability of structural elements loaded both statically and dynamically [43,44,45,46,47,48].

Unfortunately, the problem of using FA as an OPC replacement is that it significantly reduces the performance of concrete composites in the early stages of their curing [49,50]. These materials significantly delay the hydration process and the setting and hardening of the concrete composite. This limits the possibility of using this type of concrete, e.g., in prefabrication, where it is required to obtain high-strength composites after short periods of curing, i.e., several days and sometimes even a dozen or so hours [51,52,53,54,55].

Therefore, in order to minimize these negative effects and enable the use of the waste in question in a wide range of management areas, research has been undertaken to increase the early strength of the ash-filled concretes through the application of a specifically formulated nano-admixture (NA).

At this point, it should be noted that admixtures that accelerate the setting and hardening of cement composites have great potential in the concrete industry. Their application is very wide and the main advantages can be seen, e.g., in concrete prefabrication plants [32,56,57]. The use of materials of this kind allows us, among other benefits, to shorten the residence time of a “fresh” prefabricated product in the form and, consequently, to speed up the production process of a construction element [58]. Undoubtedly, such activities are particularly desirable in the industrial production of prefabricated concrete elements, which should be characterized by both high technical parameters and high repeatability of the properties of subsequent manufactured elements. Moreover, such admixtures are also effectively used in the rapidly growing and prospective 3D building printing technology [59,60,61,62].

The most popular and widely used admixture for the last few decades to accelerate the setting and hardening of concrete has been calcium chloride—CaCl_2_. This agent is very effective in accelerating the hydration of calcium silicates, mainly allite. Another advantage of this material is its low price. Unfortunately, a significant disadvantage of this admixture is its content of chloride ions, which, in the context of reinforcing steel (reinforced concrete elements), can cause its corrosion. In addition, after careful study, the negative effects of this admixture were noted as follows:

- interaction with reactive aggregates;
- increased rheology of concrete composites, i.e., shrinkage and creep;
- faster drying of the concrete mixture;
- frost resistance of the composite.

Therefore, the use of CaCl_2_ as a bonding accelerating admixture has not been recommended or even prohibited for several decades [63]. For this purpose, other chemical compounds can be used, such as calcium (III) nitrate, sodium (V) nitrate, calcium formate and sodium formate. Unfortunately, also in the case of these admixtures, apart from the undoubted benefits in the rapid catalysis of the setting processes of the cementitious composites’ structure, numerous negative effects associated with their use have been observed. Unfavorable chemical reactions of these substances with some aggregates have been pointed out. In addition, the effectiveness of some of these materials also depends on the chemical composition of the cement used, e.g., an appropriate SO_3_ content. Nevertheless, according to [64], most cements used do not meet such requirements. For these reasons, the abovementioned group of admixtures is currently not widely used for improving the curing rate of cementitious composites.

In this context, very promising results in terms of accelerating the growth of hydration products in the early and very early stages of curing of composites with cementitious matrices have been generated by the application of a nano-admixture (NA) in the form of nano-sized CSH phase crystals. The developed NA is part of a modern technological idea already successfully applied for some time, which consists in the application of nanotechnology in the construction materials industry. To date, solutions of this type have already been largely implemented in the development of nano-additives in the form of active particles of nanosilica, nanotitanium, carbon nanotubes and others [65]. A relatively new solution, however, is to use the capabilities of nanotechnology to develop an effective chemical NA.

**Figure 1:**
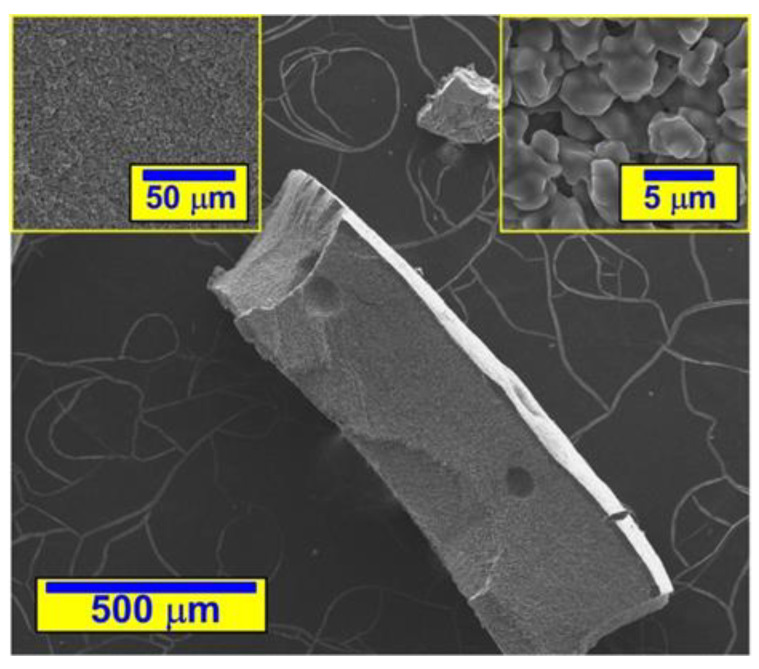
Morphology of NA in the form of CSH nanoseeds (authors’ photo).

The NA in question is an aqueous suspension of the crystallization seeds. Its morphological view by a scanning electron microscope, after drying and pulverization, along with two effective magnifications of the preparation surface, is shown in Figure 1.

The “seeding” technology was used to develop the NA. This allows the rapid growth of hydrated calcium silicate crystals in the cement paste structure, which in turn leads to the much faster growth of the final hydration products in the structure of the cement matrix containing the FA [66]. The use of CSH phase seeds as activators of early curing processes in the cement pastes leads to the construction of a microstructure with improved physical and mechanical properties, which neutralizes the FA’s negative effects on the structure of the cement matrix in the early periods of its curing.

With respect to the production of precast elements, this nano-admixture results in:

- significant acceleration of the early strength of composites;
- shortening or completely eliminating the heat treatment of prefabricated elements;
- better utilization of the prefabrication plant production capacity;
- acceleration of a production cycle;
- improvement in the formwork utilization due to earlier casting;
- increase in the efficiency of the production process;
- reduction in energy consumption;
- optimization of the binding materials used, i.e., cement and mineral additives;
- increased durability of composites.

In terms of the possibility of using the NA in sustainable concrete composites, research has been conducted to date to a rather limited extent. It has mainly consisted of evaluating the possibility of its use to improve the mechanical parameters and the structure of concretes at an early and very early age [67].

It was found, among others, that the effect of CSH nanoparticles is significant during the first day of material curing. The results of other studies show that the NA’s beneficial effect persists up to the 28th day of curing. However, in the period between the 1st and 28th day, an undoubted decrease in the effect of the increase in mechanical parameters in this type of composite is visible [68].

Additionally, it was also found that after the application of the nano-admixture, the CSH structure develops not only on the cement grains and its hydration products but also on the added seeds [68]. As demonstrated by some researchers [69,70,71,72], the range of chemical reactions causing beneficial effects in the structure of cement composites due to the application of CSH phase nano-seeds was also analyzed in considerable depth.

On the basis of a thorough review of the literature in the field of the undertaken research topic, however, it has been established that only two papers presenting the results of tests on concretes modified with the combined addition of FA and NA have been published so far. The papers, published only a year ago, presented the results of such materials from the point of view of the analysis of the chemical reactions occurring in the OPC–FA–NA mixture [73,74]. It also has been shown that it is possible to effectively substitute a cement binder with a high FA content by using active NA containing reactive particles of the CSH phase [73,74]. Apart from these papers and the authors’ earlier works regarding the rheological parameters of cement pastes containing NA [36,37,38], no research results have been encountered that would describe the relationships between the basic mechanical parameters of composites of this kind and their modified structure.

Therefore, the authors of this paper performed a series of macroscopic and microstructural experiments on the presented subject. Their aim was to determine the relations and mechanisms occurring in the properties of composites with cementitious matrices, for the fabrication of which both FA and a modern NA additive in the form of active CSH seeds were used.

The result of the undertaken research was to determine the possibility of using the combination of an active pozzolanic additive, i.e., FA, and chemically active nanoparticles with seeds of the CSH phase, i.e., NA in application for the production of sustainable precast concrete elements.

Thus, this paper presents the results of research presenting the possibility of accelerating the setting time in the FA concrete composites by means of a chemical NA in the form of nano-metric seeds of the CSH phase. The presented solution is one of the numerous examples of effective and modern industrial waste management leading to the implementation of sustainable precast composites.

The critical assessment of the proposed solution was based on the results of measurements conducted both:

- at the macro scale on concrete samples and
- at the micro scale on cement paste samples.

The results of macroscopic measurements were supported by thorough statistical analysis, whereas the microscopic examinations of composites were based on a multilevel evaluation of their structures. An attempt was made to correlate the results of the basic mechanical parameters of the composites with the morphology of the structures forming their internal skeleton.

## 2. Materials and Experimental Methodology

### 2.1. Scope of the Studies

In order to gain a thorough understanding of the relationship between the microstructure and mechanical parameters of commonly used FA-modified concrete composites, which were further modified with the modern NA with CSH seeds, an extensive laboratory measurement program was developed. Due to its orientation on composites used in precast concrete production, all experiments were carried out on concretes and cement pastes at a very early age, i.e., within the initial 72 h of their curing. It should be noted that both types of composites were made of the same raw materials and were subjected to the same regime of production and curing.

In addition, the significant properties of the two main modifiers of the composites in question, i.e., FA and NA, were characterized in the first stage of the study. For example, the crystalline phases of the FA and NA were identified by XRD patterns, which were evaluated in the presented studies.

After analyzing the raw materials, in the next step, specimens with FA and NA were prepared and used in all the basic tests. Experiments were performed for four types of composites containing a different percentage content of both FA and NA, i.e.,

- 0% FA + 0% NA (0FA0NA);
- 0% FA + 4% NA (0FA4NA);
- 20% FA + 0% NA (20FA0NA);
- 20% FA + 4% NA (20FA4NA).

Thanks to the proposed compositions of concrete and paste mixtures, it was possible to determine the NA’s influence on the properties of unmodified composites as well as mixtures in which some OPC content was substituted by FA. The FA content was assumed at the level of 20% because, as shown in previous studies, such a content of the modifier has a positive effect on numerous mechanical parameters and the structure of composites with an altered binder composition [75,76,77,78,79,80].

Although, based on the results of previous studies, it could be stated that, with the exception of concrete flatwork, FA content of up to 50% may be suitable for most elements, provided that the early age strength requirements of the project can be met and adequate moist-curing can be ensured [81,82], it was indicated by an in-depth literature review that the optimum FA content of 20–30% gave the best concrete performance in terms of substitution and addition to the cementitious composites [83,84,85,86,87,88,89,90]. Therefore, in our experiments, 20% of FA addition was used.

Basic research included the analysis of the composites’ properties of identical composition after 5 different curing periods with the following scope:

- evaluation of the strength parameters of the concrete;
- diagnostics of the cement paste structure.

In terms of strength tests, the following were evaluated in concretes:

- compressive strength *f*_cm_;
- splitting tensile strength *f*_ctm_.

**Figure 2:**
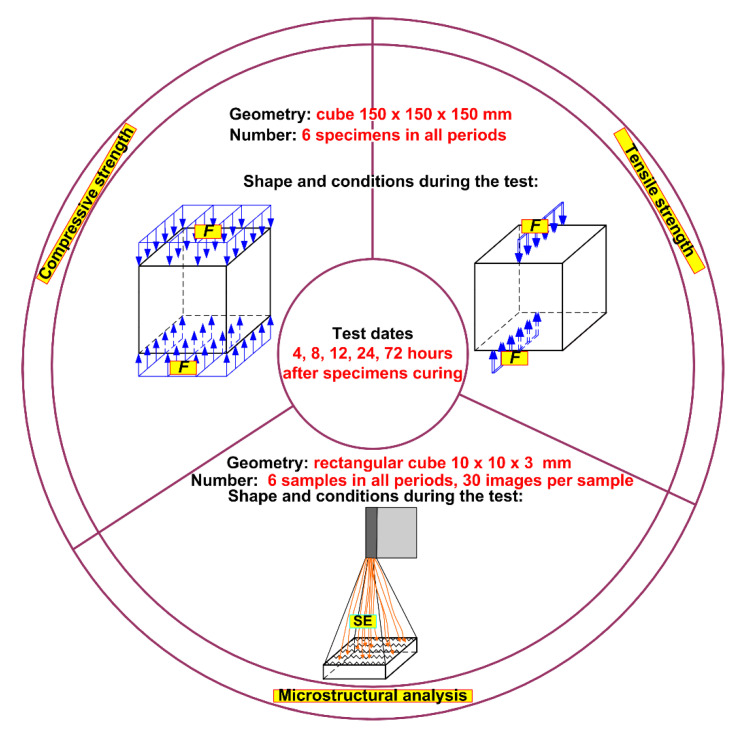
Flowchart of the study: F—force, SE—secondary electrons.

Figure 2 presents the full scope of the research program and gives the shape and geometric dimensions of the specimens used in the planned experiments. Macroscopic and microstructural tests—planned for 4 material compositions– were performed at 5 time intervals, i.e., after 4, 8, 12, 24 and 72 h. The very short experimental periods chosen in this way were dictated by matching the curing periods of the specimens at a very early age to the realities occurring in the production of precast concrete products in industrial prefabrication plants.

### 2.2. Materials

In order to prepare concrete specimens, the following materials were used:

- ordinary Portland cement (OPC) from Chełm Cement Plant;
- siliceous fly ash (FA) from Puławy thermal-electric power station;
- pit sand from Markuszów deposit;
- gravel from Las Suwalski deposit;
- nano-admixture (NA);
- the laboratory pipeline water;
- plasticizer STACHEPLAST 125;
- superplasticizer MasterGlenium ACE 430.

The OPC CEM I 32.5R with a specific surface area of 4294 cm^2^/g and bulk density of 3.22 g/cm^3^ was used to produce the concretes.

**Figure 3:**
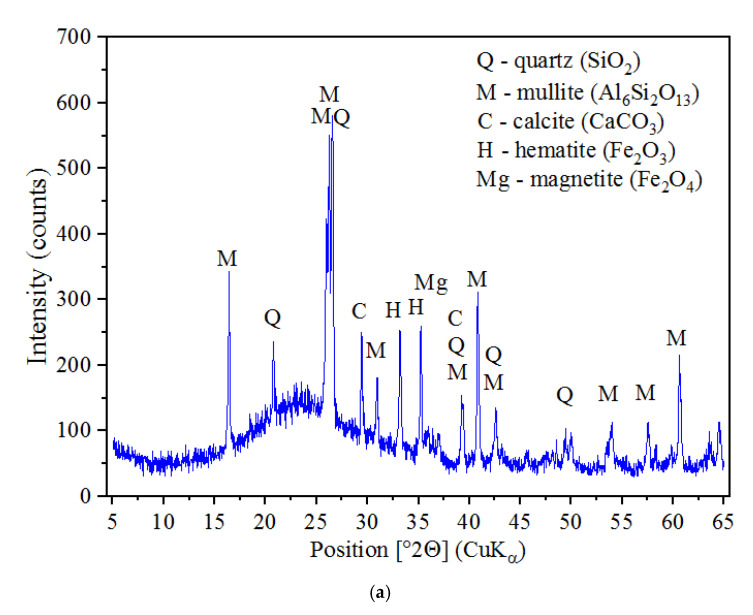

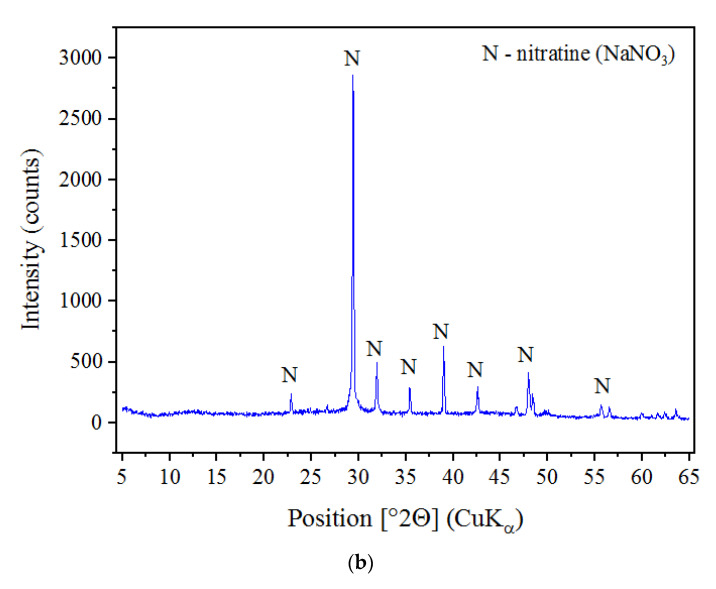
XRD patterns of the applied modifiers of cement matrix: (**a**) FA, (**b**) NA.

The FA used as a mineral additive for concretes was a waste material from the combustion processes of coal dust, from a local thermal power plant. Its mineral composition is shown in Figure 3a, whereas the main physical parameters of this waste were as follows:

- specific gravity—2.14 g/cm^3^;
- specific surface area—2944 g/cm^3^;
- fineness—39.2%;
- average particle diameter—30 μm.

Based on the results obtained, it was found that the FA applied can be an effective modifier of the structure of cement composites because:

- it is a fine-grained material consisting of particles with a low bulk density and high specific surface area;
- due to the high intensity of the peaks of its two primary phases, quartz and mullite, it can be stated that it has high pozzolanic activity (Figure 3a).

Sand of 0–2 mm fraction and gravel of 0–8 mm fraction were used in the study. Selection of aggregates with such a grain size was mainly due to their possible use in producing concrete composites, which would be applied in the production of structural prefabricated elements.

The NA containing the active CSH seeds was developed based on the proprietary Crystal Speed HardeningTM concept. Its characteristics with regard to application in the production of the precast elements is summarized in Section 1. Undoubtedly, the main advantage of the admixture is the possibility of significantly increasing the early strength of the composite, which could result in shortening the exposure time of the precast element under accelerated thermal curing conditions or even eliminating its thermal treatment. Such a property is particularly desirable for the production of precast elements composed of composites containing FA.

The mineral composition of the NA is shown in Figure 3b, while the main physical parameters of the NA are listed below:

- density of suspension—1.14 g/cm^3^;
- pH—11.5;
- chloride content—<0.1%;
- alkali content—<4.0%.

Based on the XRD analysis, it could be concluded that nitronatrite was the dominant component of the NA (Figure 3b). This mineral occurs naturally in the form of sodium nitrate (NaNO_3_).

Moreover, it should be stated that the NA applied met all the requirements for admixtures for concrete, mortars and pastes, in terms of the requirements specified in EN 934-2 + A1: 2012 [91]. Thanks to this, all the requirements for concrete mixtures prepared on the basis of the modern material, i.e., the NA, were met.

A calcium lignosulfonate-based plasticizer was used in this study with a density of 1.20 g/cm^3^ and, as a superplasticizer, the admixture with a density of 1.06 g/cm^3^ was used. In its composition, the admixture was based on a new generation of polycarboxylate ether.

### 2.3. Preparation and Casting of Test Specimens

The specimens for all tests were composed of concrete mixtures of solid materials—binder, sand, gravel, NA, water and plasticizer or superplasticizer—the content of which amounted to 352, 676, 1205, 14, 141 and 2 kg/m^3^, respectively. In individual series of concretes, only the quantity of FA and NA used were changed (see Section 3.1).

The stages of the mixing procedure included:

- mix gravel and sand in a drum mixer for several minutes;
- add the binding materials, i.e., OPC and next FA, and mix for 3 min;
- add half of the portion of water and mix for 2 min;
- add the remaining water, and plasticizer in the case of mixtures without NA, or add the remaining water, NA and superplasticizer in the case of mixtures including NA, to obtain a homogenous mixture.

The molded specimens were kept under laboratory conditions. All specimens tested within 24 h were removed from the molds immediately prior to testing. Specimens tested after 72 h were removed from the molds after 1 day and then placed in water at 20 °C ± 2 °C until the date of the planned experiments. Specimens that were cured for the longest period were removed from the water bath 1 h before the scheduled testing.

### 2.4. Test Methods

#### 2.4.1. Mechanical Parameter Analysis

The study of the basic mechanical parameters of the concretes, i.e., compressive strength and splitting tensile strength, was carried out on a testing machine with a maximum force of 3000 kN. The following assumptions were made while carrying out the experiments:

- static specimen loading process;
- control of the force increase in the specimens by increasing the displacement of the press head at 0.5 MPa/s.

#### 2.4.2. Microstructural Investigations

The microstructural analyses of the composites in question were performed by a scanning electron microscope (SEM) QUANTA FEG 250 (FEI Company; Hillsboro, OR, USA), which was equipped with an energy dispersive spectroscopy instrument (EDS EDAX) (AMETEK Inc.; Berwyn, PA, USA), on samples of specially prepared cement pastes (Figure 3).

**Figure 4:**
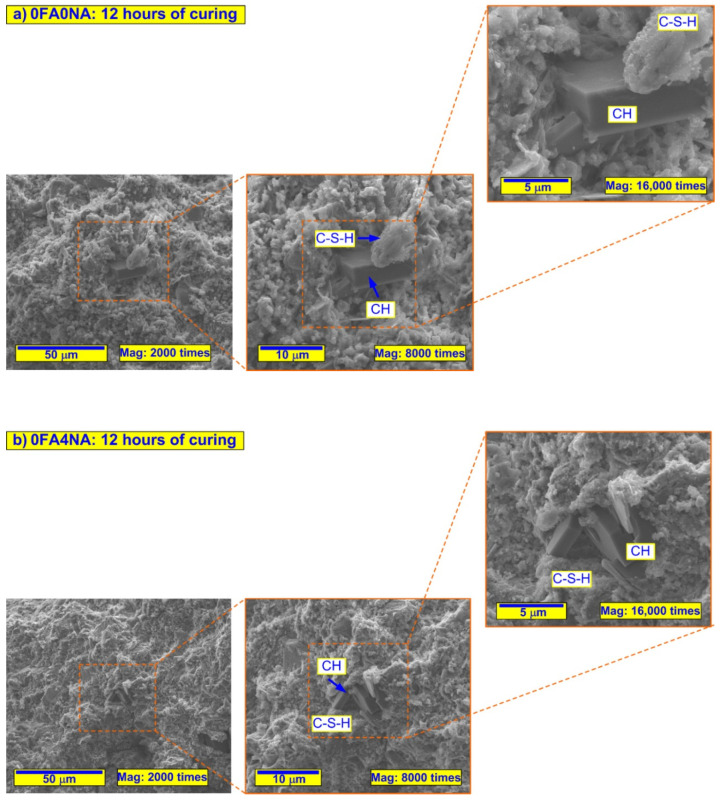

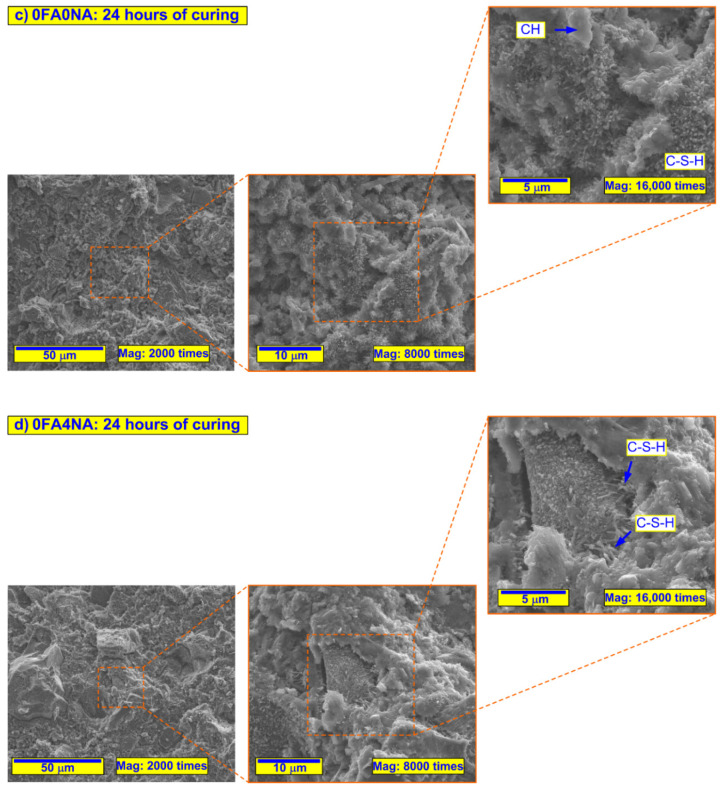

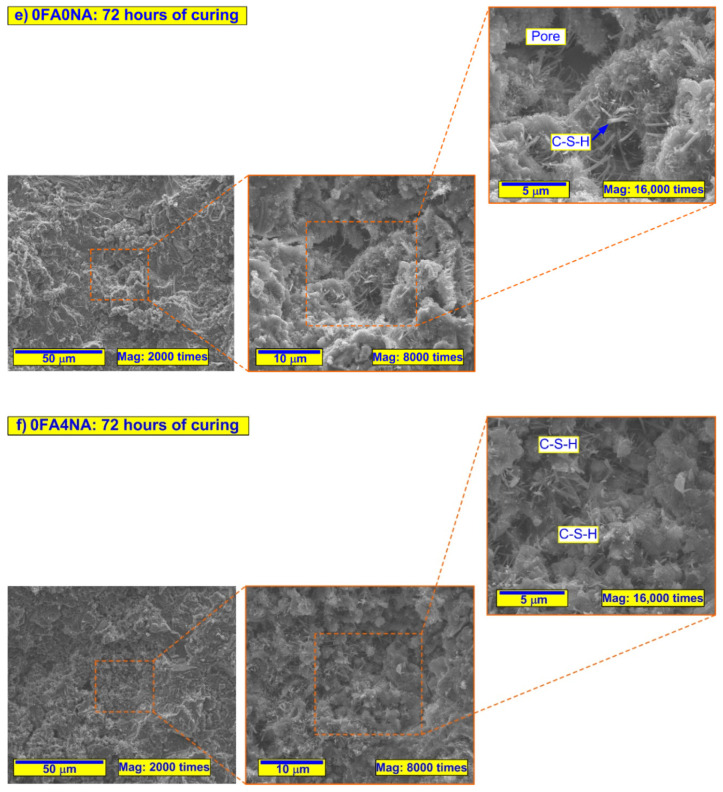
SEM micrographs of analyzed composites without FA at selected significant periods of curing: (**a**) 0FA0NA—12 h of curing, (**b**) 0FA4NA—12 h of curing, (**c**) 0FA0NA—24 h of curing, (**d**) 0FA4NA—24 h of curing, (**e**) 0FA0NA—72 h of curing, (**f**) 0FA4NA—72 h of curing.

**Figure 5:**
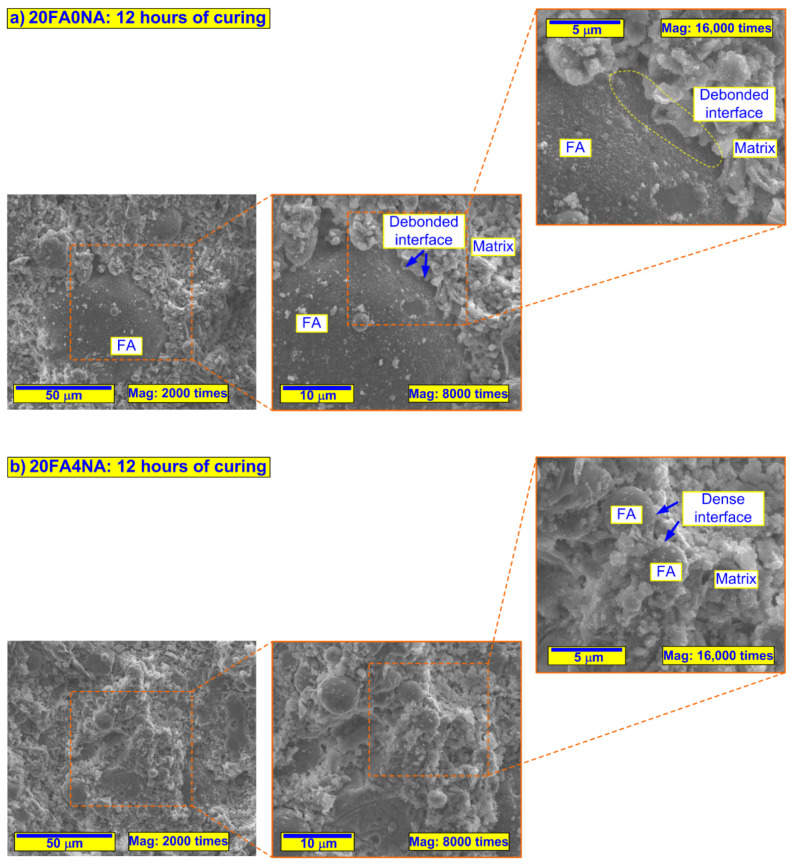

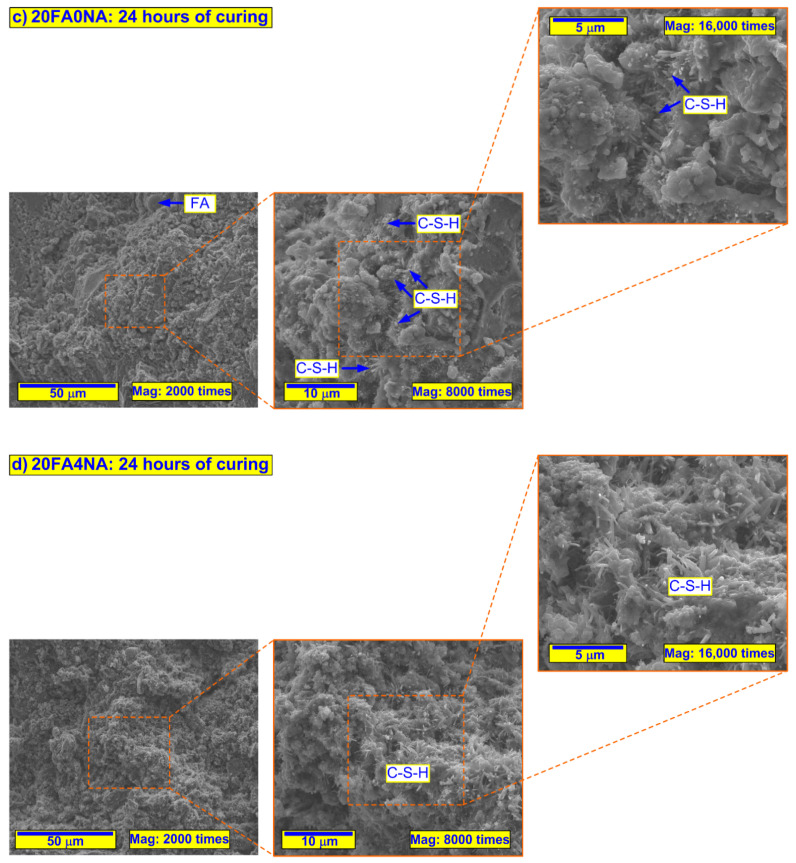

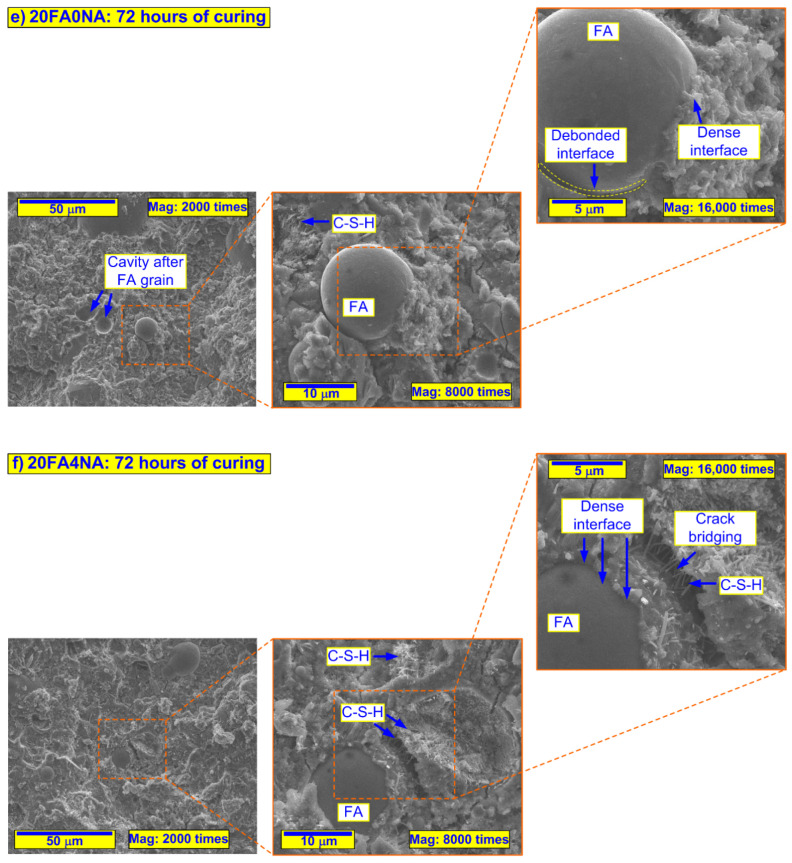
SEM micrographs of analyzed composites including FA in selected significant periods of curing: (**a**) 0FA0NA—12 h of curing, (**b**) 20FA4NA—12 h of curing, (**c**) 0FA0NA—24 h of curing, (**d**) 20FA4NA—24 h of curing, (**e**) 0FA0NA—72 h of curing, (**f**) 20FA4NA—72 h of curing.

In order to be able to accurately compare the SEM images taken, the following assumptions were made during the experiments:

- the changes in the microstructure of the analyzed composites were assessed at 3 measurement levels;
- for each of the sample, the following magnifications of structures were used: 2000, 8000 and 16,000 times;
- three reference scales, i.e., 50, 10 and 5 µm, were used to assessed each kind of composite;
- for all materials, the results were presented in the same way, i.e., by showing increasing magnifications of the selected representative area of the cement matrix;
- the structure analyses were carried out separately for the pastes containing FA (Figure 4) and the OPC-based composites (Figure 5).

During the SEM investigations, special attention was paid to the:

- type and intensity of phases in all analyzed composites;
- morphology of ITZ between FA grains and the cement matrix;
- differences in the nucleation and growth of CSH and CH phases for conventional and NA-containing composites with CSH seeds.

In addition, in order to thoroughly explain the significant differences in the basic strength parameters and microstructure of the samples containing the active C-S-H phase particles used to reinforce the composites, the formation pattern of the compact cement matrix structure during the hydration process of the OPC components was analyzed. For this purpose, additional experiments were carried out using the SEM and EDS technique—the EDAX X-ray microprobe. Thanks to the obtained results, it was possible to identify differences in the proportions of two important oxides, i.e., CaO (C) and SiO_2_ (S), in the analyzed materials. This allowed us to precisely explain the reasons for the strengthening of composites with cementitious matrices as a result of the application of the active NA particles to their structure. The method of evaluating the value of the C/S ratio was also used earlier to analyze the changes in the structure of composites modified with FA from biomass combustion [92,93].

It was estimated that by far the greatest strength of the matrix was due to the hydration of allite (C_3_S) and the subsequent formation of hydration products in the form of hydrated calcium silicates (C-S-H). The hydration of belite (C_2_S) also had a significant effect on strength, but this occurred at a later stage. The other major components of the Portland clinker, such as tricalcium aluminate (C_3_A) and brownmillerite (C_4_AF), had a much lower influence on the strength.

## 3. Results and Discussion

### 3.1. Compressive Strength f_cm_ and Splitting Tensile Strength f_ctm_

Table 1 and Table 2 summarize, respectively, the results of the compressive and splitting tensile strength tests of the analyzed concrete composites for different periods of curing.

A significant increase in *f*_cm_ and *f*_ctm_ for all mixtures containing NA with active CSH seeds was found from the compressive and splitting tensile strength testing of the concretes. Even the first organoleptic experiments related to demolding the specimens confirmed the higher compactness of concretes modified in this way. The compressive strengths of the composites without NA during the first 4 h were very low and equal to 0.32 MPa for the 0FA0NA series (Table 1). The 20FA0NA specimens could not even be demolded without failure. As in the case of the next macroscopic test, i.e., the tensile strength, this resulted in obtaining, for this series, zero values of both analyzed strengths, after 4 h of curing of the specimens (Table 2). On the other hand, significantly higher strengths were obtained in concretes with NA. In addition, similar strengths were observed for FA-modified and unmodified specimens during the first test period, i.e., 1.24 and 1.55 MPa, respectively. The strength increment in the first four hours for the FA-free mix after the NA application was therefore nearly five times. In the case of the 20FA4NA series, it was not possible to determine the level of increase because zero compressive strength was achieved in this case for the reference mix (Table 1).

A clear effect of the NA on the mechanical parameters of the composites was observed in the next testing period, i.e., after 8 h. Unfortunately, in this case, it was also not possible to demold the reference specimens containing FA for tensile strength tests without any defects. However, very high increases in *f*_cm_ were observed for concretes with a combination of FA and NA, i.e., by almost 300% (Table 2). For the composite unmodified but reinforced with the NA, the increment was almost 240% (Table 1). The highest percentage increase in both groups of analyzed parameters was also observed during this study period. In the case of the 0FA4NA series, the tensile strength equal to 1.05 MPa was as much as 650% higher than that determined for the reference concrete, which was 0.14 MPa (Table 2). A positive result of *f*_ctm_ = 0.55 MPa was also obtained for the 20FA4NA series. In fact, in the case of the 20FA0NA, the value of this parameter during this period was 0 MPa. The reason for this, as mentioned earlier, was the inability to demold the samples (Table 2).

At 12 h after specimens were formed, both *f*_cm_ and *f*_ctm_ increases for all materials were already significant. For specimens without FA, the increase in compressive strength after the application of the CSH seeds was almost two-fold, similar to that for the FA specimens (Tables).

An even more pronounced effect of strengthening the structure of composites by the use of the NA in question was observed during this research period when analyzing the values of tensile strength. This parameter was found to increase by almost 300% for composites with zero content of FA and by over 400% for concretes containing 20% of this additive (Tables).

Analyzing the next two test periods during which the basic strength parameters of the specimens were evaluated, i.e., after 24 and 72 h, the phenomenon of growth inhibition in these periods for both *f*_cm_ and *f*_ctm_ was observed. After 24 h of curing, the application of CSH phase seeds already had a less pronounced effect on the strength increments of composites both with FA and unmodified, which confirmed the results of the study by [88]. The differences between the series of reference concretes and those containing NA were insignificant and the increments of both strength parameters oscillated at the level from several to several dozen percent. Nevertheless, a clear and positive effect of the proposed material modification could still be observed.

After 72 h, the strength values of the composites with and without NA were similar. In the case of concretes made on pure OPC, 3% and 2% increases in mechanical parameters were visible for *f*_cm_ and *f*_ctm_, respectively, while, in concretes with CFA addition, decreases in both analyzed strengths were observed at the same percentage level. Thus, it could be concluded that, after 3 days, the effect of the increase in basic strength parameters in both FA and unmodified concretes disappeared.

### 3.2. Microstructure of Composites

**Figure 6:**
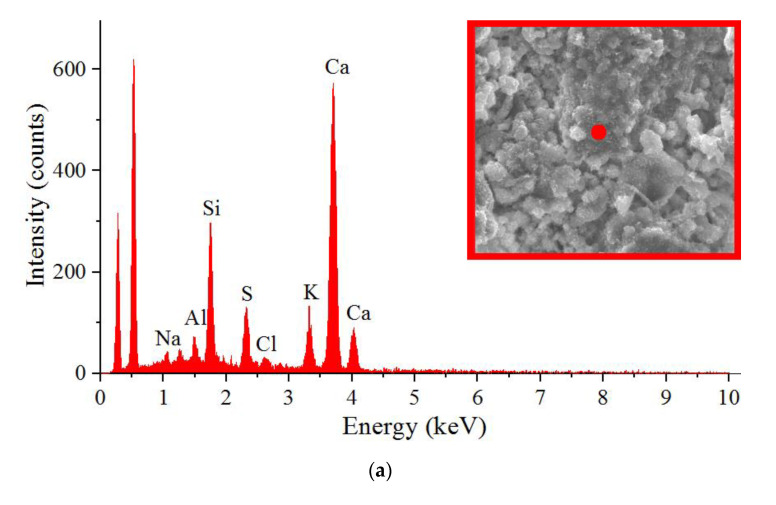

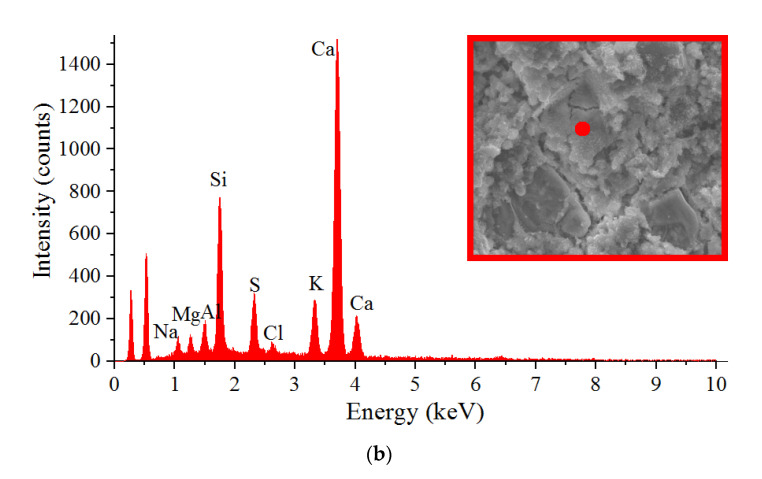
SEM–EDS results for the 20FA0NA (**a**) and 20FA4NA (**b**) after 8 h of curing.

**Figure 7:**
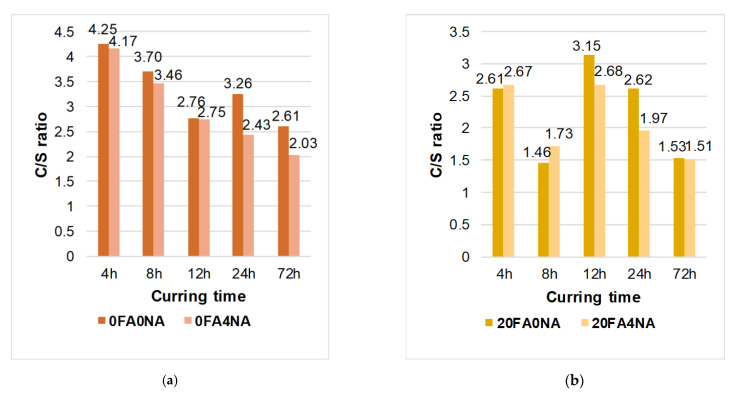
C/S ratio for analyzed samples: (**a**) without FA—relationship between 0FA4NA and 0FA0NA, (**b**) including FA—relationship between 20FA4NA and 20FA0NA.

**Figure 8:**
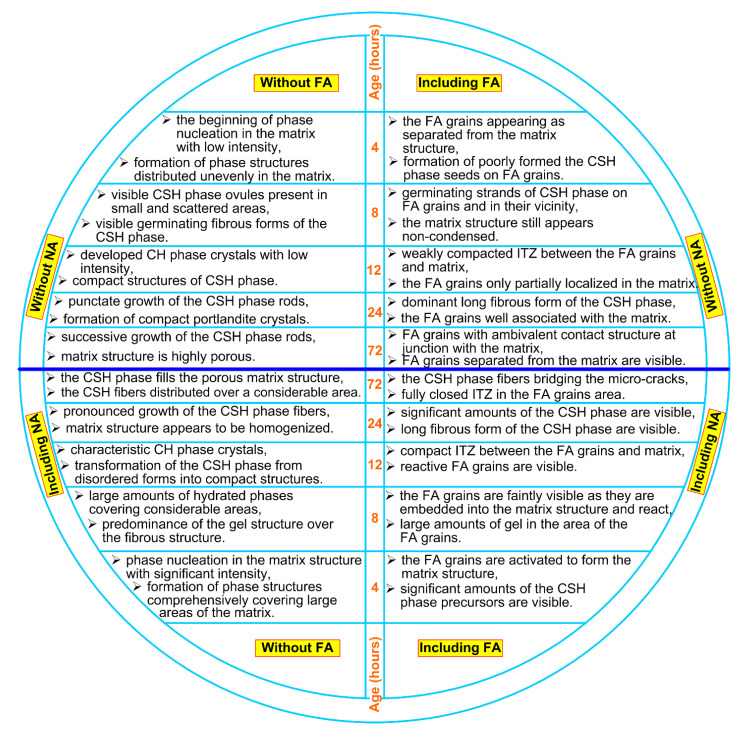
The material characteristics, features and morphology of the cement matrix, visible on SEM images of analyzed composites, at particular periods of curing.

Figure 4 and Figure 5 show sample representative SEM images of the microstructures of all the composites analyzed. Since significant differences in the structure of the analyzed materials were visible only after 12 h of curing, Figure 4 and Figure 5 show only SEM images for three time periods, i.e., 12, 24 and 72 h. Moreover, for all the time intervals, the EDS spectra were analyzed for changes in the C/S ratio values (Figure 6 and Figure 7). In addition, Figure 8 shows the differences (in a condensed format) observed in the structure of the cementitious matrix after the NA application for all the intervals studied. 

Figure 6 shows example EDS spectra for the structures of composites with FA, containing the active NA (Figure 6b) and produced without it (Figure 6a), after 8 h of curing. Figure 7 shows the comparison of the C/S ratio for the samples produced only with the cementitious binder (Figure 7a) as well as those containing 20% FA (Figure 7b) in all of the analyzed time periods.

By analyzing the SEM images and SEM–EDS spectra of the samples at successive periods, the formation of successive hydration products was evident. These products could be seen in the form of crystalline phases and the formation of portlandite plates (which are a product of allite hydration). The use of the NA (nanometric seeds of the CSH phase in liquid form) significantly contributed to the formation of more silicate gel (C-S-H) responsible for the strength of the cement matrix.

Based on the analysis of the elemental composition shown in the SEM–EDS spectrum, a lower ratio of calcium oxide and silicon oxide could be observed for the samples modified by the NA. A decrease in the C/S ratio was clearly shown, in both types of composites, after the application of the active particles of the C-S-H phase to their structure (Figure 7).

The increasing amount of silica relative to the amount of calcium indicated the degree of hydration. The lower value of the C/S ratio for samples modified with the nano-admixture indicated the faster and more efficient development of the silica gel. The C-S-H phase developed, at first, mainly on the surface of the cement grains, then filling the free spaces. The use of the NA, in the form of nanometric seeds, helped to fill the free zones between the grains of the binder (OPC and FA) and to form the product, which was the C-S-H gel. The structure of the matrix was thus tighter and more robust—which was confirmed by the microstructural analyses (Figure 5)—and thus more durable, as shown by the tests of the basic strength parameters (Tables).

Based on the conducted studies, a clear influence of the CSH seeds on the curing processes of composites could be observed. It caused the faster and more dynamic development of the cement matrix structure at a very early age. The main observations from these analyses are presented in Figure 8.

## 4. Conclusions

From this research, the following conclusions can be formulated:

1. Application of the NA with CSH seeds significantly improved the strength parameters of the FA concretes at very early ages. Several-fold increases in both analyzed strengths were observed after 4, 8 and 12 h of concrete curing. After 1 day, this effect was significantly reduced, while after 3 days, it was already negligible.
2. Nevertheless, the NA application could neutralize the negative effect of FA, which, when added alone, markedly decreased the mechanical properties of concrete composites in the early curing period.
3. The NA activity in building a compact cementitious matrix structure was observed by the thickening of its skeleton and the sealing of the free spaces (Figure 4f), which were clearly visible in composites without its inclusion (Figure 4e).
4. The FA grains in the structure of NA-containing composites entered the pozzolanic reaction faster and displayed compact contacts with the cement matrix. Their intensified reactivity resulted in the occurrence of self-healing processes in the matrix structure due to the bridging of early internal cracks.
5. The proposed NA can be an ideal solution for the management of the production of sustainable concrete and reinforced concrete prefabricated elements produced using a binder modified by FA.

## Figures and Tables

**Table 1:** Compressive strength *f*_cm_ of concretes for each curing time.

Mix	Age (Hours)	*f*_cm_ (MPa)	δ (MPa)	ν (%)	*f*_c,max_ (MPa)	*f*_c,min_ (MPa)
0FA0NA	4 h	0.32	0.01	2.95	0.33	0.30
0FA4NA	4 h	1.55	0.19	12.26	1.67	1.13
20FA0NA	4 h	0.00	0.00	0.00	0.00	0.00
20FA4NA	4 h	1.24	0.28	22.28	1.61	0.94
0FA0NA	8 h	2.06	0.13	6.22	2.24	1.81
0FA4NA	8 h	6.93	0.60	8.61	8.00	6.38
20FA0NA	8 h	0.93	0.12	13.20	1.07	0.72
20FA4NA	8 h	3.62	0.77	21.22	4.50	2.65
0FA0NA	12 h	11.80	1.34	11.37	13.65	9.40
0FA4NA	12 h	20.10	2.03	10.11	23.78	17.77
20FA0NA	12 h	3.67	0.70	19.16	4.46	2.30
20FA4NA	12 h	7.41	1.37	18.46	9.14	5.08
0FA0NA	24 h	26.87	1.27	4.71	28.31	25.14
0FA4NA	12 h	20.10	2.03	10.11	23.78	17.77
20FA0NA	24 h	16.30	2.27	13.91	20.27	13.85
20FA4NA	24 h	18.78	1.58	8.43	20.99	15.79
0FA0NA	72 h	40.41	1.18	2.91	42.03	38.60
0FA4NA	72 h	41.62	1.80	4.32	44.94	39.91
20FA0NA	72 h	29.21	0.58	1.97	30.26	28.46
20FA4NA	72 h	28.30	2.65	9.37	31.64	24.40

**Table 2:** Splitting tensile strength *f*_ctm_ of concretes for each curing time.

Mix	Age (Hours)	*f*_ctm_ (MPa)	δ (MPa)	ν (%)	*f*_ct,max_ (MPa)	*f*_ct,min_ (MPa)
0FA0NA	4 h	0.00	0.00	0.00	0.00	0.00
0FA4NA	4 h	0.24	0.02	8.17	0.28	0.22
20FA0NA	4 h	0.00	0.00	0.00	0.00	0.00
20FA4NA	4 h	0.00	0.00	0.00	0.00	0.00
0FA0NA	8 h	0.14	0.01	4.81	0.15	0.12
0FA4NA	8 h	1.05	0.20	18.99	1.29	0.76
20FA0NA	8 h	0.00	0.00	0.00	0.00	0.00
20FA4NA	8 h	0.55	0.13	24.15	0.69	0.36
0FA0NA	12 h	0.56	0.08	14.89	0.68	0.42
0FA4NA	12 h	2.16	0.16	7.52	2.44	1.95
20FA0NA	12 h	0.23	0.03	13.31	0.27	0.17
20FA4NA	12 h	1.20	0.03	2.41	1.25	1.17
0FA0NA	24 h	2.01	0.14	6.79	2.20	1.79
0FA4NA	24 h	2.40	0.21	8.57	2.74	2.20
20FA0NA	24 h	1.39	0.15	10.73	1.60	1.15
20FA4NA	24 h	2.05	0.13	6.52	2.19	1.83
0FA0NA	72 h	3.05	0.14	4.61	3.20	2.83
0FA4NA	72 h	3.12	0.22	7.02	3.42	2.82
20FA0NA	72 h	2.06	0.11	5.27	2.20	1.84
20FA4NA	72 h	2.02	0.07	3.58	2.14	1.90

## Data Availability

No new data were created or analyzed in this study. Data sharing is not applicable to this article.
